# The Oldest Representative of the Rove Beetle Tribe Pinophilini (Coleoptera: Staphylinidae: Paederinae), from Upper Cretaceous Burmese Amber

**DOI:** 10.3390/insects11030174

**Published:** 2020-03-10

**Authors:** Josh Jenkins Shaw, Bo Wang, Ming Bai, Dagmara Żyła

**Affiliations:** 1 Key Laboratory of Zoological Systematics and Evolution, Institute of Zoology, Chinese Academy of Sciences, Beijing 100101, China; joshjenkinsshaw@ioz.ac.cn; 2 State Key Laboratory of Palaeobiology and Stratigraphy, Nanjing Institute of Geology and Palaeontology and Center for Excellence in Life and Paleoenvironment, Chinese Academy of Sciences, 39 East Beijing Road, Nanjing 210008, China; bowang@nigpas.ac.cn; 3 Iowa State University, Department of Ecology, Evolution, & Organismal Biology, 2200 Osborn Dr., Bessey Hall, Ames, IA 50011, USA; zyladagmara@gmail.com; 4 University of Gdańsk, Department of Invertebrate Zoology and Parasitology, Wita Stwosza 59, 80-308 Gdańsk, Poland

**Keywords:** Procirrina, Mesozoic, amber, new genus and species

## Abstract

The recently reviewed subtribe Procirrina comprises eight extant genera with a predominately (sub)tropical distribution. Previous phylogenies consistently recover the tribe Pinophilini of the subfamily Paederinae monophyletic. No fossils of the tribe have been described, although compression fossils are known from the Cenozoic Green River Formation (50.3–46.2 Ma) as well as inclusions from the Miocene Dominican (20.43–13.65 Ma) and Mexican (20–15 Ma) ambers. Here we describe †*Cretoprocirrus trichotos* Jenkins Shaw and Żyła gen. et sp. n., the oldest fossil representative of the tribe Pinophilini, from Upper Cretaceous Burmese amber (ca. 99 Ma). Phylogenetic analyses of morphological data allow its unambiguous placement in the subtribe Procirrina. †*Cretoprocirrus trichotos* is the second genus of Paederinae described from Burmese amber and provides an important insight into the evolution of the subfamily.

## 1. Introduction

The family Staphylinidae contains over 64,000 described species in 32 subfamilies, making it the most speciose animal family known [1]. Among all subfamilies of rove beetles, the Paederinae are one of the more diverse, with 7584 described extant species and 34 fossil species [1,2,3]. Recent molecular [2], total-evidence [3] and morphological [4] phylogenetic analyses of Paederinae have contributed a wealth of data, helped to improve the classification [2] and provided the first morphological matrices for the entire subfamily [3,4]. In Żyła et al. [3], the authors also described the first representatives of the subfamily Paederinae from Burmese amber and summarized the fossil record of the subfamily. Before that, some molecular data for Paederinae was produced as a side result of larger studies focused on Coleoptera in general or Staphyliniformia and related families. Zhang and Zhou [5] included four genera of Paederinae and McKenna et al. [6] contained 11 paederine genera, two of them from the tribe Pinophilini. The latter study, however, was based on only two gene fragments [6].

The tribe Pinophilini is one of four tribes that constitute the subfamily Paederinae [2]. Pinophilini is further divided into two subtribes, namely Pinophilina and Procirrina. Pinophilini has been recovered as monophyletic with strong support based on molecular [2,6], total-evidence [3] and morphological [4] analysis, albeit based on only two species (one from each subtribe) in all of the aforementioned papers (species of *Palaminus* Erichson, 1838 and *Pinophilus* Gravenhorst, 1802 in the first paper, *Oedichirus* Erichson, 1839 and *Pinophilus* in the second and *Procirrus* Latreille, 1829 and *Pinophilus* in the last). Procirrina contains 764 species placed in eight genera [1] that are predominantly tropical in distribution [7]. Two of the most species-rich genera, *Palaminus* and *Oedichirus*, exhibit disjunct distributions between the Old and New World [7]. Species of Procirrina are typically found in leaf litter or on bushes and trees [7] and are also known from caves [8]. The genus-level taxonomy of the subtribe was treated by Herman [7], the New World species of *Oedichirus* was revised also by Herman [9] and various other revisionary works of that diverse genus e.g., [10,11] mean that Procirrina are reasonably well known. In contrast, the fossil record of Pinophilini is poorly known, although Chatzimanolis [12] reported two undescribed fossil representatives of the tribe Pinophilini from the Cenozoic Green River formation (50.3–46.2 Ma), one of which is suggested to be close to the extant genus *Palaminus*. We are also aware of more Pinophilina fossils from the same formation (D.Ż., personal observation). In addition, Seevers [13] reported a species of *Palaminus* from Oligocene/Miocene amber from Mexico, and Herman [7], personal observation therein; D.Ż. additional observation] reported at least nine specimens from the perhaps younger Dominican amber.

Although the monophyly and infra-subtribal relationships of Procirrina remain to be rigorously tested using molecular data, the description of fossil taxa and their phylogenetic placement based on morphology is important for future studies on the group. Therefore, here we describe *Cretoprocirrus trichotos* gen. et sp. n. from Upper Cretaceous Burmese amber and assess the systematic position of the new taxon based on phylogenetic analysis of morphological data using Bayesian inference and maximum parsimony.

## 2. Materials and Methods

### 2.1. Specimen, Deposition, Photography and Measurements

The beetle specimen is embedded in a medium size oval piece (32 × 26 × 5 mm) of Burmese amber from the Hukawng Valley, Kachin State, northern Myanmar and has the specimen number NIGP172214. The age of the amber is considered as Late Cretaceous (earliest Cenomanian, ca. 99 Ma) based on UePb zircon dating [14]. The specimen is quite well preserved, but observations are only possible in dorsal and ventral views. The head of the beetle is directed slightly downwards, meaning ventral head characters are not visible. The right side of the specimen (particularly from the elytra to the anterior portion of the abdomen) is partly obscured by organic matter. The holotype (NIGP172214) is deposited in the Nanjing Institute of Geology and Palaeontology (NIGP), Chinese Academy of Sciences (CAS). The specimen was studied using an Olympus SZ61 (Tokyo, Japan) stereomicroscope. Photographs were taken using a Canon EOS 5D MkII (Tokyo, Japan) with a Canon MP-E 65 mm macro lens (Tokyo, Japan) or a Zeiss AX10 microscope and Zeiss Aziocam 105 color. Photographs were stacked using Helicon Focus. X-ray micro-computed tomography (micro-CT) was attempted using an Xradia MicroXCT-400, but reconstruction was unsuccessful, probably because of lack of density contrast.

The following measurements are given (all in millimeters): head width (HW), measured from the outer margin of one eye across to the outer margin of the other; head length (HL), measured from the anterior margin of the frontoclypeus to the anterior margin of the nuchal constriction; neck width (NW), measured across the narrowest part of the nuchal constriction; pronotal length (PL), measured mid-longitudinally from the anterior to the posterior margin; pronotal width (PW), measured across the widest place, usually at about the anterior third; elytral length (EL), measured from the posterior edge of the scutellum to a line across the posterior most portion of the posterior margin of the elytra; elytral width (EW), measured across the widest place. Measurements were taken using ImageJ [15].

### 2.2. Phylogenetic Analyses

Initial examination of the fossil suggested that it was likely to be either a member of, or closely related to, the subtribe Procirrina (tribe Pinophilini) of the subfamily Paederinae. To ascertain the systematic position of the fossil, the matrix of Herman [9] was compiled in Mesquite [16] and modified according to our taxon sampling. This matrix was published as an erratum in Herman [9] but refers to the study by Herman [7] and is available at http://hdl.handle.net/2246/6421. We found it necessary to re-word some characters and their states without changing their meaning.

We added 23 more characters, some used before and some used here for the subfamily for the first time. A list of the characters studied and their respective states is provided below. To make the phylogenetic analysis more representative of the entire subfamily, seven additional taxa representing a broad spectrum of Paederinae and two additional outgroups, *Pseudopsis* Newman, 1834 (Pseudopsinae) and *Quedius molochinus* (Gravenhorst, 1806) (Staphylininae), were added. Unknown character states were coded with “?” and inapplicable states with “–”. The final matrix consisted of 57 characters scored for 21 taxa and is available as the nexus file in Supplementary Materials and in MorphoBank (project no. 3619) under this permalink http://morphobank.org/permalink/?P3619.

Bayesian inference and maximum parsimony methods were used for phylogenetic analyses to test for topological congruence.

For the Bayesian inference, MrBayes 3.2.6 [17] was used running on CIPRES Science Gateway v3.3. (phylo.org). The data were analyzed using the Mkv model [18] and default settings for priors. The analyses used four chains (one cold and three heated) and two runs of 10 million generations; they were conducted using a gamma distribution. The convergence of the two runs was visualized in Tracer v1.6 [19] and by examining the potential scale reduction factor (PSRF) values and the average standard deviation of split frequencies in the MrBayes output. Topological convergence was confirmed using the R package, RWTY (R We There Yet) [20].

The maximum parsimony (MP) analyses were conducted in TNT 1.5 [21] using the “traditional search” option to find the most parsimonious trees (MPTs) under the follow parameters: memory set to hold 999,999 trees; tree bisection–reconnection (TBR) branch-swapping algorithm with 1000 replications saving 10 trees per replicate; and zero-length branches collapsed after the search. All character states were treated as unordered and equally weighted. Node support was assessed with 1000 bootstrap (BS) pseudoreplicates.

Nodes with BI posterior probability (PP) > 0.80 or MP bootstrap (BS) values > 70 were considered well-supported; nodes with PP = 0.70–0.80 or BS = 50–70 weakly supported, and nodes with PP < 0.70 or BS < 50 were considered to be unsupported [22].

### 2.3. Morphological Characters

A single asterisk (*) indicates characters from Herman [7], two asterisk (**) characters were included in Żyła et al. [3] and Bogri et al. [4] and three asterisk (***) characters are used for the subfamily for the first time.

1*. Antennae, form: (0) straight; (1) geniculate.

2**. Head, “shelf” concealing antennal bases: (0) absent; (1) present.

3**. Antennae, antennomeres 9 and 10, shape: (0) elongated, filiform, thin; (1) elongated, egg or funnel shaped; (2) wider than long.

4*. Antennomere 11, apical spiniform pencil of setae: (0) absent; (1) present.

5*. Antennomere 11, length: (0) shorter than antennomeres 9 and 10 combined; (1) longer than antennomeres 9 and 10 combined.

6**. Eyes, setae between ommatidia: (0) absent; (1) present.

7*. Head, labrum, submedial anterior margin, development: (0) denticles and lobes absent; (1) denticles, one pair; (2) denticles, two pairs; (3) lobes, one pair.

8*. Head, epistomal suture: (0) present; (1) absent.

9***. Head, maxillary palpomere 3, shape: (0) elongated, regular; (1) expanded, more fusiform; (2) slightly expanded, vase-like.

10**. Head, maxillary palpomere 4, form: (0) parallel sided, slender; (1) nipple-like; (2) fusiform, elongate; (3) securiform; (4) truncate; (5) acicular.

11*. Head, maxillary palpomere 4, cross-section: (0) cylindrical; (1) compressed.

12*. Head, maxillary palpomere 4, pubescence: (0) absent; (1) fine and dense.

13***. Head, maxillary palpomere 4, width: (0) narrower than palpomere 3; (1) as wide or almost as wide as palpomere 3; (2) wider than palpomere 3.

14*. Head, shape (including eyes): (0) rectangular, elongate; (1) transverse; (2) elongate, strongly tapered posteriorly; (3) hexagonal.

15*. Head, pedunculate base: (0) absent; (1) present.

16**. Head, ventral side, postocular grooves: (0) absent; (1) present.

17**. Head, posterior margin, temples, shape: (0) straight; (1) rounded.

18***. Head vs. pronotum, width: (0) head narrower or as wide as pronotum; (1) head wider than pronotum.

19**. Head vs. pronotum, length: (0) head shorter or as long as pronotum; (1) head longer than pronotum.

20**. Neck, width: (0) narrow, equal or less than 1/3 of head width (1) regular, equal or less than 1/2 of head width; (2) wide, more than 1/2 of head width; (3) very wide, as wide as head.

21**. Pronotum, length: (0) longer than wide: (1) length and width subequal; (2) wider than long; (3) extremely elongated (twice as long as wide).

22**. Pronotum, widest at: (0) base; (1) apex or anterior to its middle; (2) middle; (3) same width everywhere.

23**. Pronotum, postcoxal process of hypomeron, development: (0) well developed and sclerotized; (1) translucent, somewhat flexible, or absent.

24*. Pronotum, postcoxal process of hypomeron, punctation: (0) absent; (1) present, sparse; (2) present, dense.

25*. Mesospiracular peritremes, development: (0) small; (1) enlarged.

26*. Mesospiracular peritremes, lateral margin: (0) separated from hypomeron; (1) fused to hypomeron.

27*. Procoxae, cavity: (0) open; (1) closed by mesothoracic peritreme.

28***. Mesothorax, elytra, elongated elytral base (waist): (0) absent; (1) present.

29*. Mesothorax, elytra, apicolateral angle, macroseta: (0) absent; (1) present.

30*. Mesothorax, elytra, posterior margin: (0) broadly and evenly rounded; (1) sinuate, lobed laterally or lateromedially; (2) emarginated.

31***. Mesothorax, elytra, row of setae on edge of posterior margin: (0) absent; (1) present.

32**. Protibia, comb-like rows of setae: (0) present; (1) absent.

33**. Protibia, comb-like rows of setae, number of fully developed rows: (0) two; (1) three; (2) four; (3) more than four.

34*. Protibia, ctenidial depression: (0) absent; (1) shallow; (2) deep.

35*. Protarsomeres 1 to 4, form: (0) cylindrical or dorsoventrally flattened; (1) protarsomeres 1 to 3 bulbous; (2) protarsomeres 1 to 4 bulbous.

36*. Protarsomere 5, ventral surface, pubescence: (0) absent or sparse; (1) dense.

37**. Protarsus, dense pale adhesive setae underneath: (0) present; (1) absent.

38*. Metatibia, apical ctenidium: (0) present on both sides and separated, inner comb long and outer short and with few tines; (1) present on both sides and connected or nearly connected to each other; inner comb moderately shorter than outer and with numerous tines; (2) present on one side only.

39***. Metatibia, width: (0) same along whole length; (1) apically expanded.

40**. Metatarsomere 1, length: (0) equal to or longer than metatarsomere 2; (1) shorter than metatarsomere 2.

41*. Metatarsomere 4, apical portion, length: (0) not or slightly extending beneath metatarsomere 5; (1) extending beneath metatarsomere 5.

42**. Intersegmental membrane, pattern of sclerites: (0) regular, brick-wall, sclerites hexagonal, rectangular or quadrangular; (1) irregular, sclerites rounded; (2) irregular, sclerites irregular.

43***. Intersegmental membrane, sclerites, degree of sclerotization: (0) weakly sclerotized, stronger only on sclerite edges; (1) entire sclerites strongly sclerotized.

44*. Abdominal intersegmental membrane, windows: (0) absent; (1) present.

45*. Abdominal segments III–VI, integument, imbricate macrosculpture: (0) absent; (1) present.

46*. Abdominal segment III, paratergites: (0) two pairs; (1) one pair; (2) paratergal carina present.

47*. Abdominal segments IV–VI, paratergites: (0) two pairs; (1) one pair; (2) absent.

48*. Abdominal segment VII, paratergites: (0) present, two pairs; (1) absent.

49*. Abdominal terga and sterna IV to VI: (0) separated; (1) fused.

50*. Abdominal tergum and sternum VII: (0) separated; (1) fused basally, with incision apically; (2) fused, without incision. 

51*. Tergum IX, lateroapical process, form: (0) curved, plate-like lobe; (1) elongate and tapered prong.

52*. Tergum IX, lateroapical process: (0) attached to base of IX; (1) separated from base of IX.

53*. Tergum IX, lateroapical process, apex: (0) straight; (1) ventrally curved.

54*. Terga IX and X: (0) separated; (1) fused.

55*. Sternum III, posteriorly directed sublateral carina: (0) absent; (1) present.

56**. Abdomen, sternite IV, anteromedian gland: (0) absent; (1) present.

57**. Male, aedeagus, well-developed parameres: (0) absent; (1) present.

## 3. Results

### 3.1. Phylogenetic Analyses

**Figure 1:**
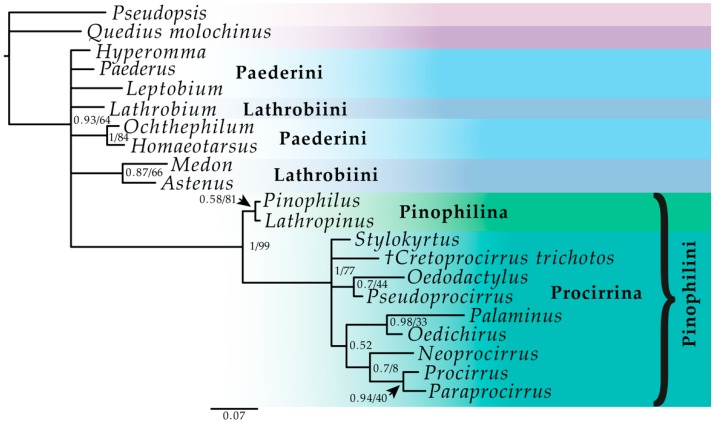
Fifty percent majority rule consensus tree from the Bayesian inference analysis of the morphological matrix. Numbers given at the nodes are posterior probabilities and bootstrap values (PP/BS).

The Bayesian inference (BI) analysis reached convergence with an average standard deviation of split frequencies well below 0.01 after 2 million generations. Most PSRF values were 1.000 (maximum 1.002). All effective sample size (ESS) values in Tracer [19] were above 200. The cumulative split frequency of all clades using RWTY [20] indicated that all chains reached convergence. We also visualized the average standard deviation of split frequencies (ASDSF) across chains as a function of chain length, which confirmed that the chains converged in their estimate of topology and support values. The maximum parsimony (MP) analysis under equal weights resulted in 68 most parsimonious trees (MPTs) with 195 steps, consistency index (CI) = 0.44 and retention index (RI) = 0.68. Overall, both BI and MP produced trees of similar topology. The placement of the fossil was the same in both. The supported topological congruence between methods is summarized on the BI tree (Figure 1). The MP strict consensus tree is provided in Supplementary Materials Figure S1.

The subfamily Paederinae was recovered as monophyletic by both methods (PP = 0.93, BS = 64). Within Paederinae, the Pinophilini were resolved as monophyletic with strong support (PP = 1, BS = 99). The relationships between the remaining Paederinae were largely unresolved. The tribes Lathrobiini and Paederini were not resolved as monophyletic by any method. Two representatives of Cryptobiina (*Ochthephilum* Stephens, 1829 and *Homaeotarsus* Hochhuth, 1851) included in our data were recovered as sister taxa with strong support (PP = 1, BS = 84). The genera *Medon* Stephens, 1833 and *Astenus* Dejean, 1833 (Lathrobiini) were recovered as sister taxa, well supported in BI (PP = 0.87) and weakly supported in MP (BS = 66) analyses. Within Pinophilini, Pinophilina represented by the genera *Pinophilus* and *Lathropinus* Sharp, 1866 was monophyletic (PP = 0.58, BS = 81), resolved as sisters to the well supported subtribe Procirrina (PP = 1, BS = 99). In Procirrina, two clades were recovered, although without support. The first recovered clade consisted of genera *Palaminus*, *Oedichirus*, *Neoprocirrus* Blackwelder, 1952, *Procirrus* and *Paraprocirrus* Bernhauer, 1923. The second included *Oedodactylus* Fairmaire and Germain, 1862 + *Pseudoprocirrus* Bernhauer, 1934, which was recovered in an unresolved position within the subtribe. The sister group relationship between *Palaminus* + *Oedichirus* was strongly supported in BI (PP = 0.98, no BS support), as was that between *Paraprocirrus* + *Procirrus* (PP = 0.94, no BS support). The positions of the genus *Stylokyrtus* Herman, 2010 and the fossil taxon described here were unresolved within the subtribe.

### 3.2. Systematic Palaeontology

Order: Coleoptera Linnaeus, 1758.

Family: Staphylinidae Latreille, 1802.

Subfamily: Paederinae Fleming, 1821.

Tribe: Pinophilini Nordmann, 1837.

Subtribe: Procirrina Bernhauer and Schubert, 1912.

**Figure 2:**
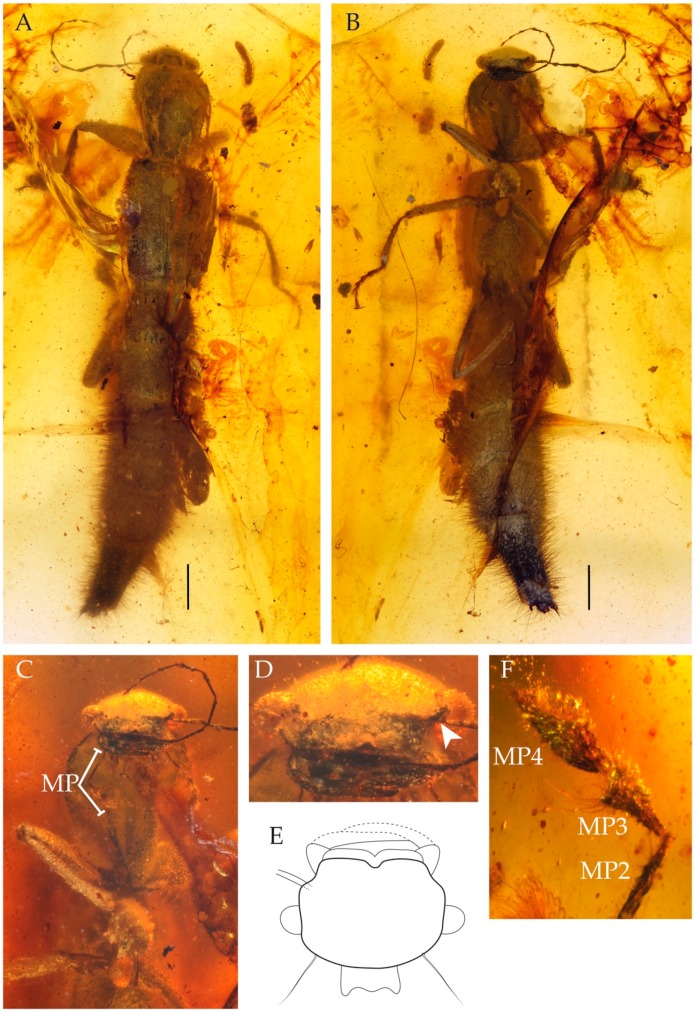
Morphology of *Cretoprocirrus trichotos* Jenkins Shaw and Żyła gen. et sp. n.: (**A**) dorsal view, (**B**) ventral view, (**C**) ventral view of forebody, (**D**) frontal view of head (white arrow = “shelf” concealing antennal bases), (**E**) reconstruction of head in dorsal view, (**F**) maxillary palpomeres 2 to 4. MP = Maxillary palpomeres. Scale bars = 1.0 mm.

**Figure 3:**
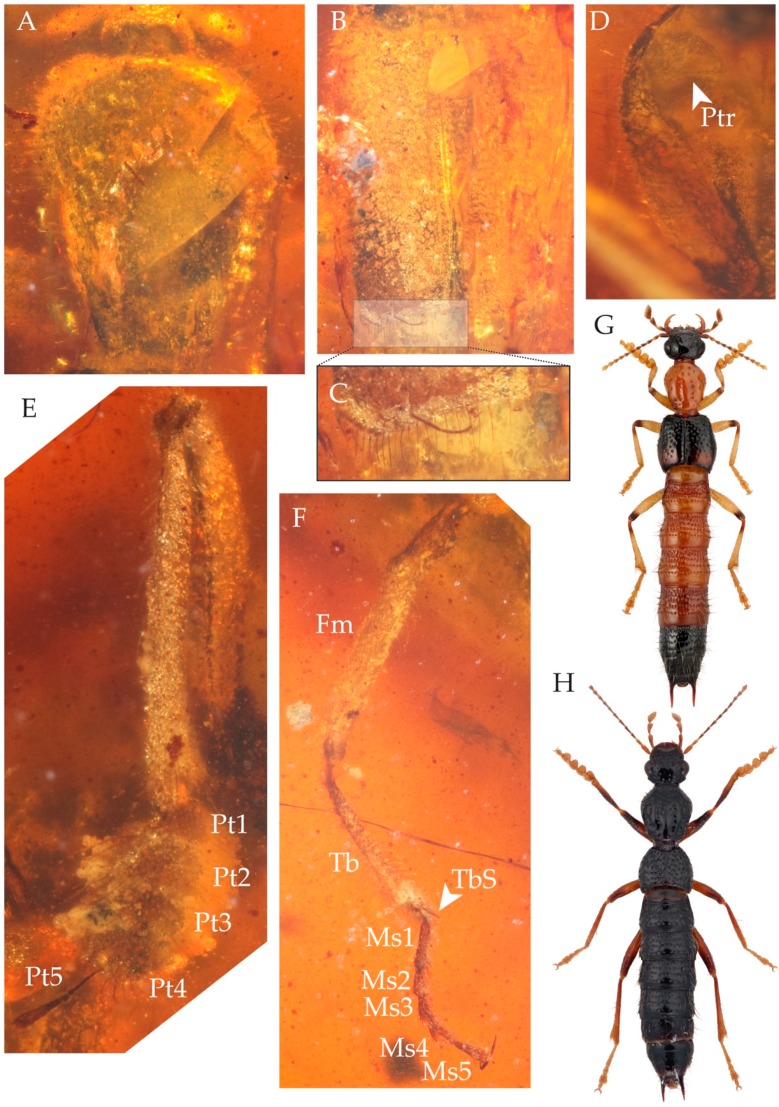
Morphology of *Cretoprocirrus trichotos* Jenkins Shaw and Żyła gen. et sp. n.: (**A**) pronotum, (**B**) elytra, (**C**) close-up of apical part of left elytron, (**D**) ventral view of left side of prothorax, (**E**) fore leg, (**F**) middle leg, (**G**) *Oedichirus lewisius* Sharp, 1874, (**H**) *Oedichirus* sp. Fm = femur, Ms = mesotarsomere, Pt = protarsomere, Ptr = protrochanter, Tb = tibia, TbS = tibial spur. Photo of *Oedichirus lewisius* from www.zin.ru\Animalia\Coleoptera (K.V. Makarov).

Genus: *Cretoprocirrus* Jenkins Shaw and Żyła gen. n. (Figure 2 and Figure 3).

Type species. *Cretoprocirrus trichotos* Jenkins Shaw and Żyła sp. n., by monotypy and present designation.

ZooBank. http://zoobank.org/urn:lsid:zoobank.org:act:CD973F06-0167-427B-B467-3246ACEBF6FA

Etymology. The genus name is derived from Cretaceous and the genus name *Procirrus*, the type genus of the subtribe.

Composition. Only type species *Cretoprocirrus trichotos* Jenkins Shaw and Żyła sp. n.

Diagnosis. Among all genera within Procirrina, *Cretoprocirrus* can be recognized based on the lack of apically expanded metatibia and presence of outer apical comb only.

*Cretoprocirrus trichotos* Jenkins Shaw and Żyła sp. n (Figure 2 and Figure 3).

ZooBank. http://zoobank.org/urn:lsid:zoobank.org:act:F6218580-D62D-4207-9A7A-C44E717D8616

Etymology. The epithet of the new species is derived from the Greek work τριχωτός (trichotós) meaning hairy and refers to the dense pubescence covering most of the body of the beetle.

Material. Holotype, female, specimen number NIGP172214 (Nanjing Institute of Geology and Palaeontology, Chinese Academy of Sciences). Most of the specimen is covered in a thin layer of air bubbles. In the dorsal view, there is a single pseudoscorpion (Arachnida: Pseudoscorpiones) to the right of the beetle.

Occurrence. Myanmar, Kachin State, Hukawng Valley; Upper Cretaceous (lowermost Cenomanian) [14].

Diagnosis. As for the genus.

Description. Large, elongate rove beetle, approximately 13 mm in length (Figure 2A,B). Measurements (in mm): HW = 1.33; NW = 0.6; PL = 2.00; PW = 1.7; EL = 2.3; EW = 1.8.

Head including eyes transverse (Figure 2C–E). Eyes distinctly bulging from side of head. Neck distinct; posterior margin strongly sinuate. Tempora with long macro seta (Figure 2E). Antennae 11 segmented; point of insertion not visible from above, concealed by “shelf” (Figure 2D); all antennomeres elongate and diminished due to preservation. Antennomere 1 about twice as long as antennomere 2. Antennomere 11 with spiniform pencil of setae apically. Maxillary palpomeres 1 and 2 elongate; maxillary palpomere 2 equal in length to 3 and 4 combined (Figure 2F); maxillary palpomere 3 expanded apically (Figure 2F), setose in apical half; maxillary palpomere 4 securiform, covered in small setae. Labial palpi: palpomere 1 not visible, palpomeres 2 and 3 equal in length; palpomere 2 with several setae apically. Frons sloping downwards anteriorly. Labrum transverse, with medial emargination, denticles and lobes absent (Figure 2E). Mandibles crossing, left mandible positioned above right mandible (Figure 2E). 

Pronotum elongate, distinctly longer than wide, widest at anterior third behind front angles (Figure 3A); sides with dense erect setae and each with single long setae, sides meeting straight base of pronotum at obtuse angle. Dorsal surface of pronotum covered in small setae. Basisternum with sharp longitudinal ridge; prosternal transverse carina acutely pointed medially.

Mesoscutellum and base of elytra deeply depressed. Elytra elongate (Figure 2A and Figure 3B); weakly tapering posteriad; laterally with dense setae; humeral angles distinct. Elytra with random punctation and area around elytral suture somewhat raised. Posterior margin of conjoined elytra emarginate, with row of setae (Figure 3C). Apicolateral setae of elytra absent. Metaventrite with several (probably four) erect macro setae in addition to usual smaller setae.

Abdomen densely covered in erect setae (some setae almost as long as length of single tergite) (Figure 2A,B); widest at segment VII. Paratergites absent, abdomen cylindrical (Figure 2A,B). Sternite III with intercoxal carina. Tergite IX with putative incomplete left lateroapical process. 

Legs relatively long. Coxae, femora and tibiae with dense erect setation. All tarsi with five tarsomeres. Protrochanter exposed, kidney shaped (Figure 3D). Profemora about as long as protibia. Inner face of protibia with distinctly longer and more densely arranged setae. Protarsomeres 1 to 4 strongly transverse (Figure 3E), ventrally with setae as well as putative adhesive setae; protarsomere 5 elongate (Figure 3E), without setae ventrally. Mesofemora and mesotibiae approximately equal in length. Mesotibiae covered in erect setae; two small spurs present apically (Figure 3F). Mesotarsomeres 1 to 4 setiferous ventrally; mesotarsomere 1 as long as 2 and 3 combined; mesotarsomere 5 approximately as long as 2 to 4 combined; mesotarsomere 4 notched apically. Metatibia of approximately equal width along entire length, not expanded apically; outer comb present. Metarsomere 1 with dense setae ventrally.

## 4. Discussion

As with many diverse organism groups, rove beetles (Coleoptera: Staphylinidae) have enormous potential for addressing questions in evolutionary biology; yet their sheer diversity acts as an impediment to their taxonomic inventory and phylogenetic comprehension. The subtribe Procirrina is just another group waiting to be explored and better understood. A quick glance at the current knowledge of the group points towards some interesting evolutionary patterns. Their predominant restriction to tropical environments, presence in arid Australia [7] and Madagascan caves [8] and the existence of two genera (*Oedichirus* and *Palaminus*) that exhibit disjunct distributions between the Old and New World are all phenomena requiring inquiry. Ideally, exploration of these should be done through fossil-calibrated dating of a molecular phylogeny or total-evidence dating. However, species level revisions and molecular data are far too incomplete to permit such research on Procirrina at present. Despite this, the description of extinct stem lineages is critical for their inclusion in future works, especially those that would address the phylogeny of Procirrina or Paederinae in a temporal context.

Burmese amber has proven to be a rich and diverse source of arthropod inclusions. As of 2018, 1192 species have been described from the deposit [23]. Among all the organisms that occur in Burmese amber, insects are the most diverse and abundant group [23]. It is unsurprising that numerous rove beetles (Staphylinidae) have been described from this deposit; with over 40 species reported so far, the number is increasing almost weekly.

Until now, within the subfamily Paederinae, just a single genus, *Diminudon* Żyła and Jenkins Shaw, 2019 with two described species, is known from Burmese amber. The genus was placed as subtribe *incertae sedis* within the tribe Lathrobiini based on a total-evidence phylogenetic analysis [3]. In the present paper we do not just describe the second genus of Paederinae from Upper Cretaceous Burmese amber, but the first fossil representative of Pinophilini and at the same time the oldest record of that group. The finding has important implications for our understanding of paederine evolution by showing that Procirrina (and therefore Pinophilini) rove beetles existed at least as far back as the Late Cretaceous.

The new species is placed in Paederinae based on the following characters, which are considered diagnostic of the subfamily [3,24]: antennal insertions concealed under “shelf” and therefore not visible from above; and hypomeron of prothorax with well-developed postcoxal process. Placement in the subtribe Procirrina is supported by the following diagnostic characters: securiform maxillary palpomere 4; protarsomeres 1–4 inflated; and abdominal segments IV to VII without paratergites [2,7]. Additional characters that support the placement in Procirrina include the long procoxae (about as long as profemora) and posterior margin of the combined elytra emarginate [7]. *Cretoprocirrus* is unusual among procirrine genera in having the metatibia of almost equal width along its entire length, not apically expanded, another character mentioned by Herman [7] that helps to define the subtribe. *Cretoprocirrus* shares with *Oedichirus* the presence of a spiniform pencil of setae on antennomere 11. Herman [7] suggested that it may be used to wick a secretion from the surface, although there is so far no evidence for this. Our phylogenetic analysis confidently places the fossil species within the subtribe Procirrina; however, its exact position remains unresolved. With the addition of our new genus, Procirrina now contains nine genera (*Procirrus*, *Neoprocirrus*, *Oedichirus, Oedodactylus*, *Palaminus*, *Paraprocirrus*, *Pseudoprocirrus*, *Stylokyrtus* and *Cretoprocirrus*; Figure 3G,H).

Burmese amber is thought to be the product of a coniferous tree belonging to the Cupressaceae or Araucariaceae, which existed in a moist tropical environment [25]. The precise paleoenvironmental conditions of the Burmese amber forest have not yet been fully established, but palynological study is indicative of a humid, warm, temperate climate [26]. Further evidence of Burmese amber formation in a tropical environment is provided by the presence of distinctly tropical taxa [25]. As mentioned by Herman [7], Procirrina are typically diverse in the tropical and subtropical regions of the world. Therefore, the presence of an extinct lineage of Procirrina from the Upper Cretaceous Burmese amber is not surprising.

With the description of *Cretoprocirrus trichotos* here, it is apparent that Paederinae rove beetles had diversified (or at least had begun to), into their major groups by the late Cretaceous. In addition to the taxon described here and *Diminudon* [3], we are aware of other, potentially distantly related paederines, in Burmese amber, which await description and phylogenetic placement.

## 5. Conclusions

*Cretoprocirrus trichotos* Jenkins Shaw and Żyła gen. et sp. n. is the first formally described fossil species belonging to the rove beetle tribe Pinophilini of the subfamily Paederinae. Based on phylogenetic analyses of a morphological dataset, the new genus is confidently placed in the subtribe Procirrina. Its discovery in the Upper Cretaceous amber along with the earlier mentioned Cenozoic and Miocene fossils [7,12,13] indicate a relatively long evolutionary history of this group dating back to the Mesozoic, with a high likelihood of more fossils waiting to be discovered.

## Supplementary Materials

The following are available online at https://www.mdpi.com/2075-4450/11/3/174/s1, File S1: Morphological matrix nexus file, Figure S1: Strict consensus tree from the maximum parsimony analysis with bootstrap values at the respective nodes.
