# Supplementary figures and images for: The Oldest Representative of the Rove Beetle Tribe Pinophilini (Coleoptera: Staphylinidae: Paederinae), from Upper Cretaceous Burmese Amber
Source: Insects. 2020 Mar 10;11(3):174. doi: 10.3390/insects11030174 (PMC7142758; doi:10.3390/insects11030174)

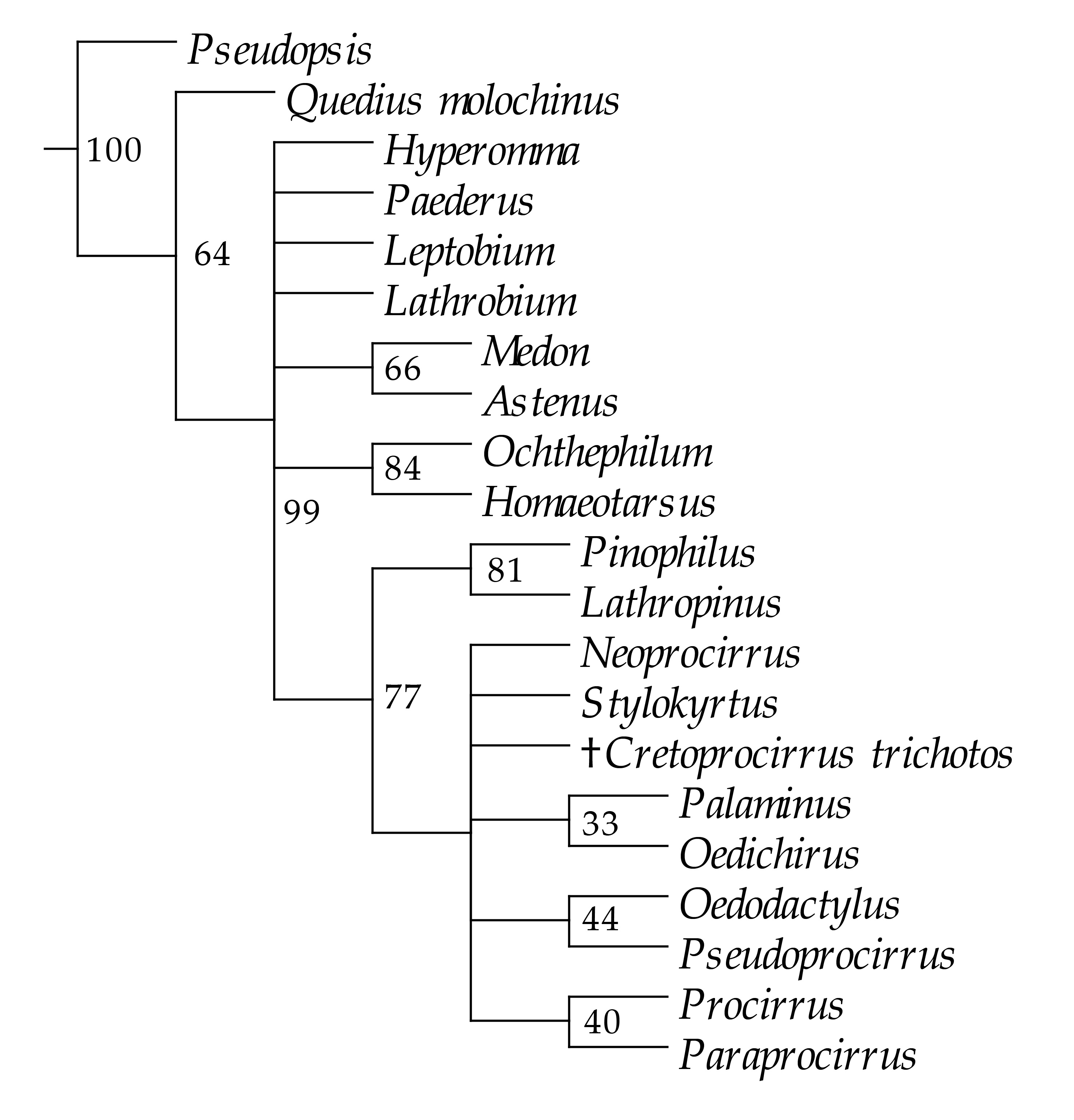

Supplement: Supplementary file 1 [file insects-11-00174-s001.zip › Figure S2-01.jpg]
